# Editorial: Recent advances of drug resistance research in colorectal cancer therapy

**DOI:** 10.3389/fonc.2022.1059223

**Published:** 2022-11-22

**Authors:** Chenyang Ye, Yanhong Deng, Weijing Sun, Ying Yuan

**Affiliations:** ^1^ Department of Medical Oncology, Key Laboratory of Cancer Prevention and Intervention, The Second Affiliated Hospital of Zhejiang University School of Medicine, Hangzhou, Zhejiang, China; ^2^ Cancer Center, Zhejiang University, Hangzhou, Zhejiang, China; ^3^ Department of Medical Oncology, Hospital of Sun Yat-sen University, Guangzhou, Guangdong, China; ^4^ Division of Medical Oncology, University of Kansas School of Medicine, Kansas, KS, United States

**Keywords:** drug resistance (DR), colorectal cancer, cancer treatment, drug resistance mechanism, prognosis analysis

Colorectal cancer (CRC) remains one of the most diagnosed and fatal cancers in China and worldwide, bringing clinical and economic burdens. Despite recent improvements in diagnosis and treatment, only approximately 20% of patients with newly diagnosed metastatic CRC survive more than 5 years. The development of drug resistance towards chemotherapy, targeted therapy, and immunotherapy is one of the major challenges in the treatment of advanced CRC. With the aid of various new biological technologies including genome editing and liquid biopsy etc., many ongoing studies aim to strengthen our understanding of the mechanisms of drug resistance and will decipher this issue in the near future.

This Research Topic brings together seven articles reviewing recent advances in the development of drug resistance in advanced CRC treatment with a different focus from basic, translational, and clinical fields. It is hoped that the collection will facilitate understanding of novel biomarkers and underlying mechanisms as well as new strategies to overcome the development of drug resistance.

Zhao et al. performed a bibliometric and visualized analysis to present the current state and changing trends of drug resistance in CRC over the past 20 years. In their paper, CiteSpace and VOSviewer were used. They found the number of publications in the field of drug resistance in CRC has increased annually. The keywords “Oxaliplatin resistance”, “microRNA” and “EMT” showed up with high frequency, which is noteworthy for future studies. Taken together, this bibliometric and visualized study comprehensively summarized the global research status of drug resistance in CRC during 2002-2021 and could facilitate understanding of the current status and future trends of drug resistance research in CRC.

Zhang et al. conducted a basic research study that investigated the role of phase II detoxifying enzyme Glutathione S-transferase alpha 4 (GSTA4) in CRC. In this study, CRISPR/Cas9 editing method was used to inactivate GSTA4 in human CRC cells. The results indicated that loss of GSTA4 significantly suppresses proliferation and clonogenicity. Importantly, the inactivation of GSTA4 increased cell susceptibility to 5-FU and oxaliplatin. Interestingly, exposure of GSTA4 loss cells to 5-FU could upregulate the expression of γH2AX, a hallmark of double-stranded DNA breaks. However, there is no increased expression of γH2AX observed in oxaliplatin-treated GSTA4 loss cells, which requires further investigation to decipher the discrepancy. This study suggests that GSTA4 promotes proliferation, tumorigenesis, and chemoresistance of CRC and could serve as a potential therapeutic target for CRC.

Tao et al. previously proved that semaphorin 3F (SEMA3F) signaling could help overcome chemotherapy resistance in CRC cells by upregulating E-cadherin and integrin αvβ3 expression levels. In light of the results from another study, indicating that upregulation of p27 could induce the expression of E-cadherin and integrin, this follow-up study aimed to assess the effect of SEMA3F on P27 and whether SEMA3F could overcome chemotherapy resistance in CRC cells *via* regulating P27. The results indicated that SEMA3F could reverse chemotherapy resistance by mediating P27’s subcellular localization in CRC cells. This study presents a novel mechanism that provides insights into the role of SEMA3F in regulating the chemosensitivity of CRC, suggesting a promising therapeutic target for CRC in further research.

High serum uric acid (SUA) levels indicate poor prognosis in digestive cancers. However, the correlation between SUA level and clinical outcomes of postoperative CRC patients treated by chemotherapy remains unknown. The retrospective study by Zhang et al. aimed to explore the relationship between baseline SUA level and progression-free survival (PFS), disease control rate (DCR), and safety in postoperative CRC patients receiving FOLFOX, FOLFIRI or XELOX treatment. Their findings suggest that high SUA levels could serve as a promising biomarker linked to worse PFS, DCR, and safety of postoperative CRC patients treated with FOLFOX or FOLFIRI. Zhang et al.’s work is the first study to investigate the links between baseline SUA levels and the prognosis, effectiveness, and safety in CRC patients who received chemotherapy following surgery. Their work implies that the baseline SUA is a promising biomarker that could indicate the clinical prognosis, DCR, and safety of postoperative chemotherapy for CRC patients.

The study conducted by Qiu et al. aimed to explore the underlying mechanism of the Traditional Chinese Medicine formula Wenzi Jiedu Recipe (WJR) in the treatment of CRC. They found that WJR exerts anti-CRC therapeutic effects both *in vivo* and *in vitro*. It is noteworthy that the results indicated that WJR could significantly upregulate the proportion of CD8+T cells and the expression of immune-associated cytokines. In addition, analysis of the gut microbiota suggested that WJR was significantly enriched for Oscillibacter and Bacteroides_acidifacien. Qiu et al.’s study investigated the merits of WJR in CRC treatment and implied the underlying mechanism of how WJR exhibited anti-cancer effects, suggesting traditional Chinese medicine formulas could also play important roles in treating CRC.

Autophagy is a physiological process that recycles the unnecessary or dysfunctional components of cells through a lysosome-dependent regulated mechanism. Autophagy not only plays a key role in maintaining metabolism and cellular homeostasis but is also involved in cancer development and progression. A paper from Manzoor et al. summarizes the role of autophagy in the tumorigenesis and drug resistance of CRC. This review focuses on the potential correlation between autophagy, tumor microenvironment, epithelial-mesenchymal transition, and drug resistance, suggesting future directions for research in this field.

T-cell exhaustion is one of the reasons for low response rates to immunotherapy in microsatellite-stable CRC. Deciphering these T-cell exhaustion-activating mechanisms could help improve the effects of immunotherapy. To this end, Paladhi et al. identified that thymidine phosphorylase (TYMP) could induce systemic T-cell exhaustion. Targeting TYMP with tipiracil hydrochloride (TPI) could lead to immunological cell death. Targeting TYMP also improved the efficacy of dendritic cell immunotherapy in CRC in mouse models. In summary, this paper provides a promising treatment strategy for promoting anti-cancer immunity.

## Author contributions

CY drafted the manuscript. WS, YD, and YY revised the manuscript and all authors approved the submission.

## Acknowledgments

We appreciate all submissions to this Research Topic and would like to thank the contributing authors and reviewers.

## Conflict of interest

The authors declare that the research was conducted in the absence of any commercial or financial relationships that could be construed as a potential conflict of interest.

## Publisher’s note

All claims expressed in this article are solely those of the authors and do not necessarily represent those of their affiliated organizations, or those of the publisher, the editors and the reviewers. Any product that may be evaluated in this article, or claim that may be made by its manufacturer, is not guaranteed or endorsed by the publisher.

